# Socio-demographic correlates of availability of adequate iodine in household salt: a community-based cross-sectional study

**DOI:** 10.1186/s13104-020-04983-w

**Published:** 2020-03-04

**Authors:** Dessalegn Ajema, Muluken Bekele, Manaye Yihune, Hiwot Tadesse, Gebrekiros Gebremichael, Melkamu Merid Mengesha

**Affiliations:** 1 Department of Public Health, College of Medicine and Health Sciences, Arba Minch University, Arba Minch, Ethiopia; 2 Department of Nursing, College of Medicine and Health Sciences, Arba Minch University, Arba Minch, Ethiopia; 3 School of Public Health, College of Health and Medical Sciences, Haramaya University, Harar, Ethiopia

**Keywords:** Iodine availability, Household salt, Prevalence, Associated factors, South Ethiopia

## Abstract

**Objective:**

This study aimed to assess the availability of adequately iodized salt at a household level and associated factors in Arba Minch town, South Ethiopia using the gold standard technique, the iodometric titration.

**Results:**

41.8% (95% CI (confidence interval) 38.6 to 45.1) of households had inadequately iodized salt, and 9.3% (95% CI 7.5 to 11.4) had an iodine content below 10 ppm (parts per million). Compared to households with a monthly income of greater than 2000 ETB (Ethiopian Birr), households with a monthly income between 1000 ETB to 2000 ETB (adjusted odds ratio (AOR) = 0.52, 95% CI 0.390.36 to 00.77) and main food handlers aged 30 years or above compared to those aged less than 20 years of age (aOR = 0.55, 95% CI 0.34 to 0.91) had higher odds of having adequately iodized salt. Food handler’s knowledge and practice were not found to be correlated with the availability of adequately iodized salt in household salt.

## Introduction

Iodine deficiency, one of the most prevalent micronutrient deficiencies globally with two billion individuals estimated to have an insufficient iodine intake [1, 2], is the main cause of potentially preventable cognitive disability in childhood [3]. When iodine requirements go unmet, synthesis of thyroid hormones is impaired, resulting in a spectrum of growth, developmental and functional abnormalities referred to as iodine deficiency disorders (IDD) [4]. Iodine deficiency most affected people in sub-Saharan Africa and Southeast Asia [2].

Globally, there have been major achievements to prevent iodine deficiency between 2003 and 2011, the number of countries with adequate iodine intake increased from 67 to 105 [5]. Despite these gains, sharp regional differences persist [5].

In Ethiopia, for the past six decades, IDDs has been recognized as a serious public health problem. An estimated 66 million people had an insufficient iodine intake and remain unprotected from iodine deficiency in 2011, with only 15% of households had access to adequately iodized salt [6]. Despite the government of Ethiopia planned to achieve 90% of households use adequately iodized salt by the year 2015, only 25.8% of households succeeded, and hence iodine deficiency has continued to be a critical public health problem in Ethiopia [7].

Only 12.2% of the households were using iodized salt in the Southern nation, nationalities, and peoples region (SNNPR), which is quite below the national level [8]. Evidence on the availability of adequately iodized salt at the household level, in areas located over 1000 km from the production site, is not well studied in a large sample using the gold standard iodometric titration [9].

## Main text

### Methods

#### Study design and population

A community-based cross-sectional study was conducted in Arba Minch town in June 2017. Study participants were primary food handlers, a household member who is mostly involved in food preparation and aged 18 years old or more. Primary food handlers who were sick or unable to respond were excluded from the study.

#### Sample size determination and sampling procedures

The sample size was calculated using Epi Info version 7 considering different parameters: 95% level of confidence, magnitude of adequately iodized salt = 33% [10], 4% margin of error, 1.5 design effect, and 10% non-response rate. Accordingly, the minimum calculated sample size was 875.

A multistage sampling technique was used to select households included in the study setting. At the first stage, we randomly selected four of the 11 kebeles (the lowest administrative unit) in the town, and households within each of the selected kebeles, in turn, are selected using a systematic random sampling technique.

#### Data collection and quality management

A structured questionnaire adapted from the iodized salt program assessment tool was used to conduct a face-to-face interview [11]. Using a plastic bag, 20-g sample (2–3 teaspoons) of salt was collected from each household and the iodine level was determined using iodometric titration in Arba Minch University biochemistry laboratory under quality control measures.

#### Procedure to test the iodine content of salt

A 10 g iodized salt is dissolved in 50 ml distilled water after ensuring the sample salt was thoroughly mixed in zip-lock bags. Once the salt is dissolved in the measured amount of water, 2 ml sulfuric acid and 5 ml potassium iodide are added to the salt solution, which in the presence of iodine, will turn yellow. The reaction mixture was then kept in a dark place (with no exposure to light) for 10 min to reach the optimal reaction time before titrated with sodium thiosulfate using starch (2 ml) as the indirect indicator [8]. Adequately iodized salt at household level was defined when a salt sample has ≥ 15 parts per million (PPM) of iodine [12].

#### Knowledge about iodized salt

Five questions assessed knowledge, and participants who scored above the median in the overall knowledge score were considered as having good knowledge and poor otherwise.

#### The practice of household salt handling

There were eight practice questions and participants who scored above the median for the practice questions on household salt handling were considered as having good practice and poor otherwise.

#### Data management and analysis procedures

Data were entered into Epi info version 7, and then exported to STATA version 14.2 for cleaning and data analysis. Results were presented using descriptive summary statistics for continuous variables and percentages for categorical variables and also presented in a diagram and in tables. Pearson’s Chi square test and a one-way Analysis Of Variance (ANOVA) were used to see if there was an association between two categorical variables and variation in mean iodine level by level of a factor variable, respectively. The binary logistic regression model was used to identify factors associated with the level of iodine in household salt. Finally, variables with P < 0.05 in the multivariable analysis were considered as significant.

### Results

#### Socio-demographic characteristics

A total of 875 subjects, representing a 100% response rate, participated in this study, of whom, males constituted only 6.3%. The mean age was 31.1 years (Table 1).

**Table 1:** Socio-demographic characteristics of respondents in Arba Minch town, Gamo Zone, South Ethiopia, 2017

Variables	Frequency	Percent (%)
Sex		
Female	820	93.7
Male	55	6.3
Age category		
< 19	106	12.1
20–29	351	40.1
30–29	218	24.9
40–49	121	13.8
50+	79	9.0
Age, mean (95% CI)	31.1 (30.4, 31.8)	
Religion		
Protestant	415	47.4
Orthodox	360	41.1
Muslim	100	11.4
Educational status		
Diploma or higher	335	38.3
Do not read and write	235	26.9
Grade 9–12	159	18.2
Grade 1–8	146	16.7
Occupation		
Private business	300	34.3
Student	183	20.9
Government employee	165	18.9
Merchant	129	14.7
Farmer/or housewife	98	11.2
Marital status		
Married	561	64.1
Single	261	29.9
Widowed	27	3.1
Divorced	26	3.0
Household monthly income		
< 1000 ETB	384	43.9
1000–2000 ETB	198	22.6
> 2000 ETB	293	33.5
Household family size		
< 5	658	75.2
≥ 5	217	24.8

#### Level of adequately iodized salt in household salt

The proportion of households with adequate iodine content in household salt was 58.2% (95% CI 54.5 to 61.4). Of the households with inadequately iodized salt, 41.8% (95% CI 38.6 to 45.1), 9.2% (95% CI 7.5 to 11.4) had an iodine content below 10 ppm. The overall mean level of iodine content in a household sample of salt was 16.4 ppm (± 5.4).

**Fig. 1:**
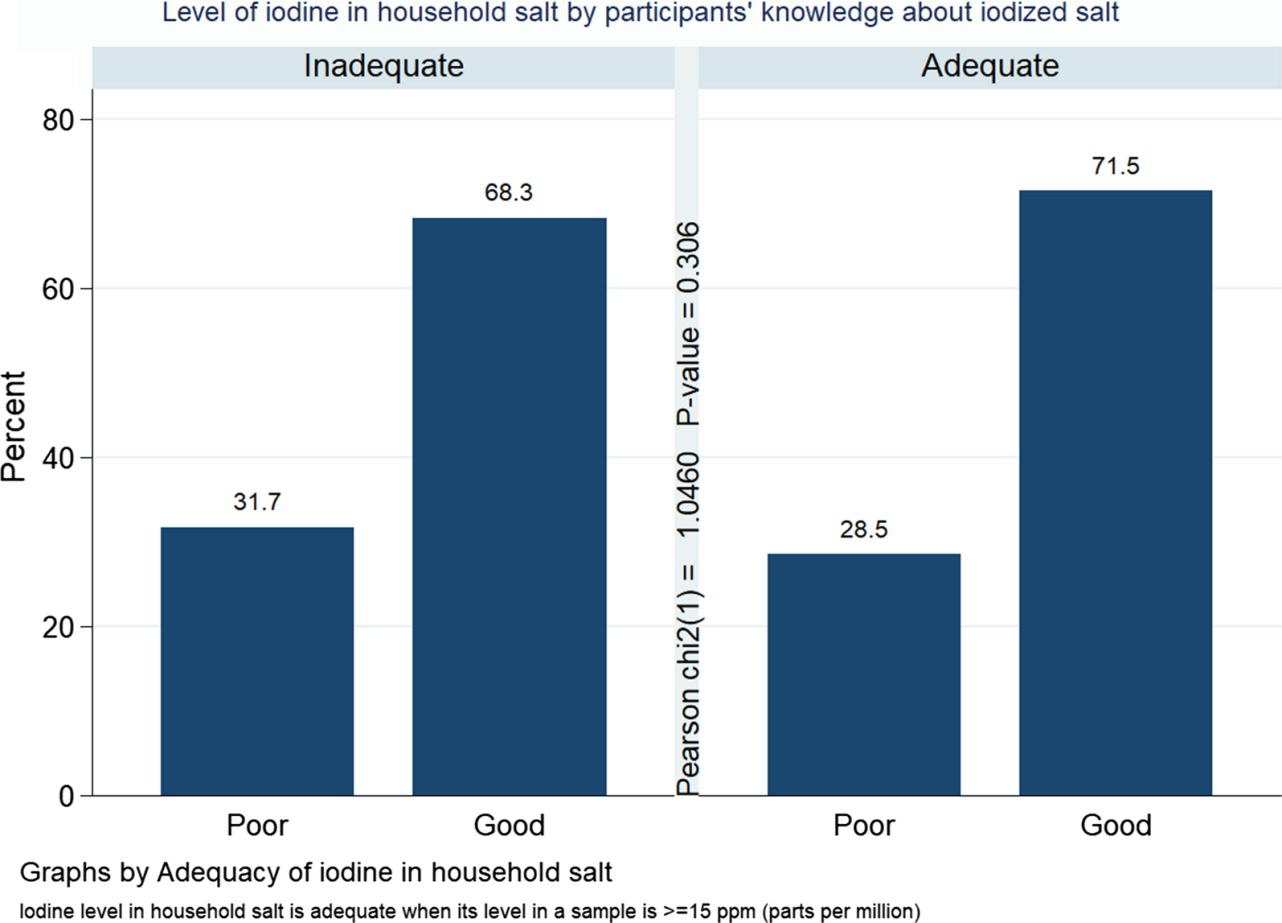
Household salt Iodine level by participants’ knowledge in Arba Minch town, Gamo Zone, South Ethiopia, 2017

There was a comparable level of knowledge about iodized salt among participants in households with inadequate versus adequate iodine content in salt, 68.3% versus 71.5%. There was no significant difference in the level of household iodine content by participant’s knowledge (Fig. 1).

#### Knowledge about iodized salt

Seventy percent of the participants reported that they had heard about iodized salt before this survey, and 26.2% did not know the negative effects associated with iodine deficiency. The commonly mentioned effect of iodine deficiency was goiter, among 60% of respondents. Regarding the benefits of iodized salt, 5.2% said it is used just for a flavor, and 33.6% reported they do not know its benefit. Similarly, 49.7% said unfavorable transport or storage of iodized salt reduces its content.

#### The practice of iodized salt utilization

The majority of respondents practiced appropriate handling of iodized salt concerning avoiding exposure to sunlight (84%), placing it in a dry area (94.2%), and storing it in a covered container (72.7%). However, 55.0% buy unpacked salt, 27.3% buy from an open market, 35.5% store for 2 or 3 months, and 55.1% add salt early or in the middle of cooking time which altogether contribute to the consumption of reduced level of iodine.

The mean level of iodine in household salt which was obtained from an open market versus a retailer’s shop or a supermarket was 15.3 ppm and 16.8 ppm which had a significant difference (F (1873) = 13.11, P-value < 0.001), respectively. Similarly, the mean level of iodine significantly varied in household salt which was packed (17.2 ppm) versus unpacked (15.8 ppm), (F (1873) = 16.34, P-value < 0.001).

#### Factors associated with adequate iodine level in household salt

There were increased odds that adequately iodized salt would be available in households where the main food handler was Orthodox compared to a Protestant by religion (aOR (adjusted odds ratio) = 1.50, 95% CI 1.10 to 2.03). However, there were reduced odds that adequately iodized salt would be available in households where the main food handler was older than 30 years (aOR = 0.50, 95% CI 0.30 to 0.85) compared to those under 20 years of age and in households where monthly income was between 1000 ETB to 2000 ETB compared those earning greater than 2000 ETB (aOR = 0.58, 95% CI 0.39 to 0.86) (Table 2).

**Table 2:** Analysis of factors associated with availability of adequately iodized salt in Arba Minch town, Gamo Zone, South Ethiopia, 2017

Variables	Level of iodine in a sample of household salt		COR, 95% CI	AOR, 95% CI
	≥ 15 ppm	< 15 ppm		
Sex				
Female	475	345	0. 85 (0.49, 1.49)	
Male	34	21	Ref.	
Age category				
< 19	70	36	Ref.	Ref.
20–29	209	142	0.71 (0.45, 1.14)	0. 74 (0.46, 1.20)
30 +	230	188	0.62 (0.39, 0.98)*	0. 55 (0.34, 0.91)*
Educational status				
Do not read and write	126	109	0.83 (0.59, 1.16)	1. 11 (0.74, 1.67)
Grade 1–8	89	57	1.12 (0.75, 1.67)	1. 34 (0.88, 2.07)
Grade 9–12	99	60	1.18 (0.80, 1.74)	1. 24 (0.83, 1.86)
Diploma or higher	195	140	Ref.	Ref.
Occupation				
Farmer/or housewife	47	51	0.61 (0.37, 1.00)	
Merchant	80	49	1.08 (0.68, 1.72)	
Government employee	96	69	0.92 (0.60, 1.42)	
Private business	176	124	0.94 (0.65, 1.37)	
Student	110	73	Ref.	
Marital status				
Married	326	235	0.91 (0.51, 1.62)	
Single	151	110	0.90 (0.49, 1.65)	
Widowed/divorced	32	21	Ref.	
Religion				
Ethiopian orthodox	232	128	1.61 (1.20, 2.15)**	1. 68 (1.25, 2.27)**
Muslim	57	43	1.17 (0.76, 1.82)	1. 16 (0.73, 1.84)
Protestant	220	195	Ref.	Ref.
Household monthly income				
< 1000 ETB	228	156	0.85 (0.62, 1.17)	0. 78 (0.55, 1.10)
1000–2000 ETB	96	102	0.55 (0.38, 0.79)**	0. 52 (0.36, 0.77)**
> 2000 ETB	185	108	Ref.	Ref.
Household family size				
< 5	389	269	1.17 (0.86, 1.59)	
≥ 5	120	97	Ref.	
Respondent’s knowledge				
Poor	145	116	Ref.	Ref.
Good	364	250	0.86 (0.64, 1.15)	1.14 (0.78, 1.66)
Respondent’s practice				
Poor	205	145	Ref.	Ref.
Good	304	221	1.03 (0.78, 1.35)	0.93 (0.67, 1.29)

* P-value < 0.05 ** P-value < 0.01

## Discussion

The proportion of households with adequately iodized salt at the national level in Ethiopia was only 25.8%, with a great sub-national variation [7]. We found that only 58.2% of households had adequately iodized salt.

The estimated proportion of households with an adequate level of iodine in Arba Minch town, 52.8%, was higher compared to the sub-national estimate for SNNPR, 13.7% [7], and also compared to a study in Wolaita zone, 37.7% [9]. Our sample, however, was restricted to households located in an urban area, and different studies reported a higher level of iodine content in urban than rural areas [9, 13–15]. Consistent with our finding, a study in southwest Ethiopia, Dera district, reported that 57.4% of households had an adequately iodized salt (≥ 15 ppm) [16]. Compared to our finding, Mekonnen et al. reported a lower proportion of households with adequately iodized salt in Kombolcha town, 35%, and a higher level in Dessie, 78% [15].

The proportion of households consuming adequately iodized salt in the study setting, 58.2%, was very low compared to the WHO recommendation of > 90% [17]. Though iodine can be obtained from several food sources, iodine-rich foods are not available everywhere [2], and hence consumption of iodized salt is the mainstay of preventing IDDs [2]. Research evidence indicated that IDD can be manifested across the life span [17] which in turn signifies the importance of reducing iodine deficiency.

We found that household monthly income and age and religion of primary food handler had a significant association with the level of iodine in household salt. Similar findings were reported in previous studies [14–16, 18, 19]. Inadequate iodine content was more likely in households where a primary food handler was of age 30 years or more as reported in previous studies [16, 19]. As we found that there was variation in iodine content by religion, a similar finding was reported by Sen et al. where consumption of adequately iodized salt was lower among Muslims [18].

Against our expectation, the respondent’s knowledge of iodized salt and handling practices had no statistically significant association with the availability of adequate iodine in household salt. A previous similar study in Addis Ababa, however, reported that level of the knowledge does not affect the availability of adequately iodized salt in household salt [20]. We justify our finding by fact that, in the year when this study was conducted, several firms were producing and distributing salt to consumers with unregulated quality [21], and hence access to mixed quality of iodized salt may underestimate the effect of consumer’s knowledge about iodized salt and handling practice in maintaining iodine in household salt. A study by Mekonnen et al. in South Wollo, Ethiopia, identified firms that distribute table salt with no iodine or inadequate iodine [15]. Similarly, substantial loss of iodine content of salt in the supply chain as high as 57% from production was also reported [22].

### Limitation of the study

The limitations of our study could be that we did not address the level of iodine in salt at the production site or in a retailer’s shop where households purchase. Furthermore, as our study was limited only to the urban areas, our results were not generalizable to households in rural areas. Furthermore, social desirability bias could be another limitation of this study.

## Data Availability

The datasets used and analyzed during the current study will be available from the corresponding author on a reasonable request.

## Abbreviations

ANOVA: Analysis of variance
EDHS: Ethiopian demographic and health survey
ICCIDD: International Council for the Control of Iodine Deficiency Disorders
IDD: Iodine deficiency disorders
IGN: Iodine global network
IQ: Intelligence quotient
PPM: Parts per million
SNNPR: Southern nation, nationalities, and peoples region
UNICEF: United Nations Children’s Fund
USI: Universal salt iodization
WHO: World Health Organization
